# A critically appraised topic (CAT) to compare the effects of single and multi-cat housing on physiological and behavioural measures of stress in domestic cats in confined environments

**DOI:** 10.1186/1746-6148-10-73

**Published:** 2014-03-22

**Authors:** Lauren R Finka, Sarah LH Ellis, Jenny Stavisky

**Affiliations:** 1 School of Life Sciences, Faculty of Science, University of Lincoln, Riseholme Park, Lincoln, Lincolnshire LN2 2LG, UK; 2 Centre for Evidence-based Veterinary Medicine, School of Veterinary Medicine and Science, The University of Nottingham, Sutton Bonington Campus, Loughborough LE12 5RD, UK

**Keywords:** Cat, Feline, Stress, Housing, Welfare

## Abstract

**Background:**

Domestic cats have evolved from solitary, asocial predators and whilst they may display social behaviours, they can still exist as solitary survivors. Over-population and relinquishment of pet cats are ubiquitous problems worldwide, and rehoming centres (also known as rescues/ shelters) aim to ameliorate this by holding cats in confinement for a variable period until a new home is found. The provision of optimal housing for large numbers of cats in close confinement, such as in rehoming centres, is therefore inherently difficult. Under these conditions there is the potential for individuals to develop signs of physical and psychological ill health, and thus experience compromised welfare. Available information regarding housing practices that maximise welfare currently provides conflicting results, and as a consequence there are no unanimous housing recommendations. The aim of this study was therefore to review the evidence on the impact of single housing compared to multi-cat housing on stress in confined cats, as measured by physiological and/or behavioural outcomes. The review was conducted using a Critically Appraised Topic (CAT) format. A systematic search of electronic databases (CAB Abstracts, Zoological Records and Medline) was carried out to identify peer-reviewed literature comparing single and multi-cat housing in confined environments.

**Results:**

A total of 959 papers were initially identified, six of which met sufficient criteria based on their relevance to be included within this review. All of the studies had significant limitations in design and methodology, including a lack of information on how groups were assigned, inconsistent handling and enrichment provision between groups, and lack of information on the socialisation status of cats.

**Conclusions:**

Whilst some studies suggested that single housing may be less stressful for cats, others suggested group housing was less stressful. Several other important factors were however identified as potential mediators of stress within the different housing systems, and recommendations based upon these findings are presented.

## Background

### Clinical scenario

Many cats are kept in captive environments such as rehoming centres (also referred to as rescues/shelters), often for indefinite periods of time. In trying to accommodate these individuals as optimally as possible, it is important that they are provided with suitable housing conditions, which aim to minimise exposure to stress in order to maximise welfare.

Whilst recommendations for the housing of the domestic cat in laboratories, rehoming centres and other facilities have been put forward [1-3], the strength of evidence in support of these recommendations is rarely considered critically, and can be contradictory. This study was conceived as part of the development of evidence-based guidelines on the housing of cats in such contained environments, specifically cat rehoming centres. The aim was to assess the evidence on whether housing cats singly as compared to groups of two or more in these types of environments results in changes to physiological and/or behavioural measures of stress, and therefore which system should be recommended as preferable in order to minimise stress.

### Introduction

A recent survey of cat rehoming organisations within the UK estimated their total intake of cats over a 12 month period to be 156,826, and 70% of these organisations were usually or always operating at full capacity [4]. Unfortunately, the provision of optimal housing for such large quantities of cats within these environments is inherently difficult, and under such conditions there is the potential for individuals to develop signs of physical and psychological ill health.

As a species, *Felis catus* is thought to have originated from primarily solitary dwelling felids [5-7], and whilst populations of free living *F. catus* may reside in groups, they may also live independently [8-11]. The feline social system is therefore one of variability and flexibility. In cat colonies, social structuring, relationships and potential conflicts may be the result of complex interactions between age, gender, sex ratio, relatedness and individuality [12]. It is thought that the occurrence of group living and the subsequent population densities of free ranging cats are ultimately influenced by the abundance of food resources rather than an inherent need for protection or regular social contact/interaction *per se*[13-17]. In contrast to free ranging populations, group living in domestic companion cats may often take the form of temporary or transitory housing during a stay in a rehoming centre, or when living in a domestic home environment. In both contexts, individuals may have limited choice or control over the nature of their ‘group living’, especially when their environment prevents them from making the choice to live independently (for example, multiple cats kept in a single enclosure at a rehoming facility, or multiple cats kept strictly indoors in the home).

It is likely that most rehoming centres will contain diverse populations of cats of varied ages and temperaments. Some cats may be related or familiar with each other (which may facilitate more amicable relationships in certain instances [18]), but the majority are potentially unrelated and also unfamiliar. For many individuals, being forced to reside in close proximity to other cats under these types of conditions may result in stress, conflict and potentially compromised health and welfare [19,20]. Organisations caring for such animals often operate under conditions of limited resources of space, staffing, time and finances. Currently, there is conflict in which housing practices are recommended to maximise use of resources but simultaneously preserve a basic standard of welfare for the cats.

The aim of this study was therefore to review the evidence on the impact of single housing compared to multi-cat housing on stress in cats, as measured by physiological or behavioural effects.

### Focussed clinical question

In [cats kept in confined environments] does [single housing compared to multi-cat housing] result in [changes in physiological and/or behavioural measures of stress]?

## Methods

### Search strategy

The search strategy included the use of three separate electronic databases; CAB Abstracts (1910 – present, via the Ovid interface), Zoological Records (1998 – 2007) and Medline (In-process & other non-indexed citations, 1946- present, via the Ovid interface). The search was conducted in October 2012.

After accounting for specific syntax associated with each database, each search had similar components (search terms are listed in Additional file 1) and all were searched as both keywords and subject heading terms, joined using Boolean operators. All references obtained were imported into Endnote, combined into a master database, and all duplicates (identified based on title, date published and authors) were removed.

### Inclusion criteria

Studies were not excluded on any grounds of quality, only on relevance to the study aim. For inclusion, papers had to include:

• Domestic cats kept in an enclosed area from which they were unable to exit (omitting the domestic home), for example, rehoming centres, boarding catteries and laboratories.

• Comparison of both single and multi-cat (i.e. two or more cats) housing conditions within a single study, with outcome measures that were either behavioural, physiological or both, and were classed as indicators of stress. Our working definition of stress was:

• “an inferred internal state which denotes a real or perceived perturbation to an organism’s physiological homeostasis or psychological well-being”, as used by Ward *et al.*[21], and similar to that used by McEwen [22], as we felt it was appropriate to this context. However many other definitions exist [23-25], and in the present study papers were not included or excluded on the basis of this definition.

• Original observed or experimental data.

Studies were also required to be peer-reviewed, with the full text available in English.

### Screening process

Two stages of eligibility screening were carried out. The first stage was completed independently by two of the authors (LF and JS), and any references that clearly did not fit the eligibility criteria were excluded. After this, in stage two, the remaining references were screened again by all three authors. For this stage, full text was retrieved for any papers where the information contained within the abstract was deemed insufficient to make a decision upon eligibility. Where there was initial disagreement over eligibility, the papers were read and discussed until consensus was reached among the reviewers [26].

### Critical appraisal

All remaining papers were independently appraised by all three authors, using critical appraisal tools developed by the Department for Emergency Medicine at Manchester Royal Infirmary (http://www.bestbets.org/) and used extensively in the literature [27-29]. These appraisals were then collated by the lead author (LF) into a summary table. All three authors re-checked this summary of evidence for consistency of interpretation.

## Results

**Figure 1:**
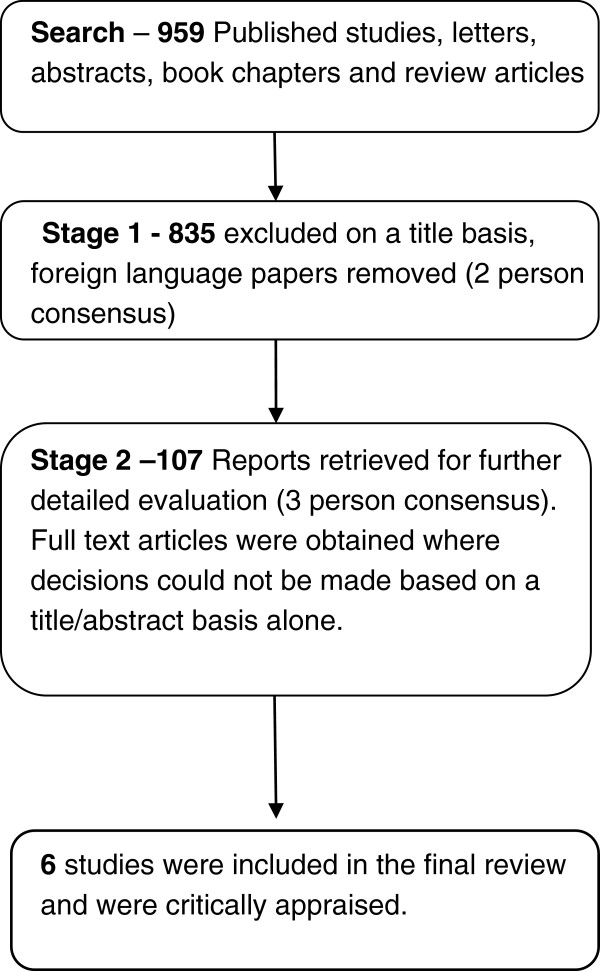
Results of searches and screening processes used to identify relevant papers.

959 papers were initially identified. Following screening as in Figure 1, six papers fulfilled all of the inclusion criteria. The results of the appraisal can be seen in Table 1.

**Table 1:** Summary of appraisal of the six papers meeting the inclusion criteria of assessing single versus multi-cat environments on physiological and behavioural measures of stress in confined domestic cats

**Author, date and title**	**Uetake and others **[30]	**Lichtsteiner and Turner **[31]*	**Gourkow N, Fraser D **[34]	**Ottway, D. S. & Hawkins, D. M. **[33]*	**Kessler M. R. & Turner D C. **[35]	**Kessler M. R, & Turner D C. **[32]
	Effects of single caging and cage size on behavior and stress level of domestic neutered cats housed in an animal shelter [30]	Influence of indoor-cat group size and dominance rank on urinary cortisol levels [31]	The effect of housing and handling practices on the welfare, behaviour and selection of domestic cats (*felis silvestris catus*) by adopters in an animal shelter [34]	Cat housing in rescue shelters: A welfare comparison between communal and discrete-unit housing [33]	Socialization and stress in cats (*felis silvestris catus*) housed singly and in groups in animal shelters [35]	Stress and adaptation of cats (*felis silvestris catus*) housed singly, in pairs and in groups in boarding catteries [32]
**Study design**	Randomised controlled trial	Controlled trial	Randomised controlled trial	Cohort	Randomised controlled trial	Cohort
**Stated aim of paper**	• To provide information on the minimum spatial requirement for singly caged cats in animal shelters	• The relevant aim was to determine “whether the urinary cortisol levels of the cats are related to environmental parameters…additionally the cortisol levels of cats from private households were compared with shelter cats to check for an influence of location”	• To examine how different housing and handling conditions affected the welfare, behaviour, adoption rate and selection of individual cats by adopters	• To test the hypothesis that, in long-term shelter care, cats housed communally with unfamiliar conspecifics experience higher levels of stress than do cats housed in discrete units, due to inappropriate and unstable social grouping	• To provide recommendation for the most suitable housing type for cats with known socialization status	• To investigate levels of stress in cats housed singly, in pairs and in groups
						• To compare stress levels in newly arrived cats to a longer-term control group
**Subjects**	• 6 cats between 2–15 years old residing in an animal shelter	• Twenty-one shelter cats	• 165 cats entering an animal shelter	• 74 cats residing in 2 animal shelters, randomly selected from the shelter population	• 169 cats between 1–8 years old residing within an animal shelter	• 140 cats between 1–15 years old, residing in a boarding cattery in 2 categories, plus a “control” group of 45 un-owned cats
		• All cats had lived in the shelter for at least 3 weeks and were considered “adoptable”	• Inclusion criteria: mixed breed, 1–7 years of age, neutered, healthy			
			• Excluded: feral cats			
				• Excluded: cats having been in the shelter < 1 month		
						• Excluded: ill cats, “highly stressed” cats (in “control” group only)
**Environment prior to study**	• All cats had previously been kept in a socially stable group environment for at least 7 months	• All cats had been in the shelter for at least 3 weeks	• Cats were from both stray and domestic home environments (numbers of each not specified)	• Cat were from both stray and domestic home environments (numbers of each not specified) and had been in the study environment for a least 1 month	• All cats were relinquished/unwanted (no history of previous long-term living experiences)	• 140 cats were owned, from single or multi-cat homes
		• Single housed cats were transferred to single housing at least one week before sampling				
	• No history of background prior to this 7 month period					
						• Origin of 45 “control” shelter cats not specified.
					• Cats in the single cage condition had previously been housed singly for 10–20 days in the study environment	
			• The study commenced on day one of exposure to the study environment for all individuals			
						• 85-87% of the owned cats had been exposed to the study environment on a previous separate occasion
					Cats in the group condition had previously been housed in a group with changing compositions for 10–20 days in the study environment	
						• Control cats had spent between 2 and 16 weeks in the study environment
**Intervention/group definition**	• All individuals were exposed either to small, medium or large single cages in varying orders, all without human social contact. This was compared with their baseline stress levels when previously group housed (it is assumed the group size at this point was six)	• Two groups, comprising six and seven cats housed communally	• Assigned to one of four housing conditions:	• In one shelter, individuals were already housed communally in one of three groups (either 33, 47 or 65 individuals per group)	• Cats housed in individual units or in a group enclosure (specific group sizes unspecified but at least >5)	• Boarding cats housed singly (60), in pairs (40) or groups (40) (each group size unspecified but at least >2) according to owner preference
			- basic single (minimal human interaction)			
		• Four of these group-housed cats were removed from each group and housed singly for one week prior to being sampled				
			- enriched single (with consistent human handling and human interaction)			
			- basic communal (eight cats per group), with consistent human handling and human interaction			
						• “Control” cats (45) were living in six groups (size unspecified), which had been stable and un-altered for at least 2 weeks prior to the study
				• In the other, 12 cats were housed in pairs and nine cats were divided into threes. These cats were previously socialised together or siblings. Additionally, 15 cats were housed singly		
			- enriched communal (eight cats per group), with consistent human handling and human interaction and extra hiding places and toys			
**Outcome measures (refer to Table**2**for further information on measures)**	• Urinary cortisol: creatinine ratios	• Urine cortisol: creatinine ratio on a single voided sample	• Cat Stress Scores (CSS)	• CSS scores and time budgets (including eat, drink, groom, play, rest, stereotypic behaviour and agonistic encounters)	• CSS	• CSS taken 4 x daily
					• Human-Approach-Test (HAT), Cat-Approach-Test (CAT) and socialisation questionnaire used to determine whether cats were socialised towards conspecifics or humans	
			• Outcome of stay i.e. adopted, not adopted, euthanized or isolated for physical health reasons			
	• Behavioural time budgets (including resting, drinking, eliminating, vacuum behaviour, and others, locomotion, social/solitary play, exploring and self-grooming)					
			• Time to adoption			
**Data collection period and frequency of relevant measures taken to assess stress.**	• Cats were exposed to each condition for six days, i.e. the study period was a total of 18 days	• A single urine sample was taken for each cat on a convenience basis during the study period (14 days).	• The study period lasted 21 days	• Data on individuals was collected each day over 15 consecutive week days, however, it is unclear if all cats were sampled for full duration due to cat turnover during the study period	• Data collected over a 7 day period	• Data collected over 14 days
			• Cats were observed for 2 minutes each day, and assigned a Cat Stress Score for the first 10 days of the study period, however, not all cats were assessed for the full 10 days			
					• Cat-Stress-Score was assessed every l0 minutes during the first hour post placement into the test condition, then twice (within a 15-min interval) after 6 hours. For the following days, 2 observations were made in the morning and 2 in the evening	• CSS were initially recorded after the first two hours of entry into the test environment and were then taken 4 times daily, each day, twice in the morning and twice in the evening
	• Behavioural observations made over 3 hours during the last 2 days in each of the different housing conditions					
	• Urine samples were collected in the morning and evening of each day and then averaged, repeated each day of the study period					
				• Cats instantaneously scan sampled and assigned a CSS each day, every 30 minutes from 08:30 am to 15:30. All other behavioural data was collected via one–zero sampling in between each scan interval		
					• HAT and CAT randomly assessed twice a day for 4 days across the test population, once in the morning and once in the evening	
**Key results**	• Time spent in locomotion and solitary play were lower in individual cages than in group housing conditions	• Urinary cortisol:creatinine ratios were highly variable	• CSS were highest in the basic single housing treatment	• No differences in CSS scores were found between cats housed individually and those housed with either one or two other familiar cats	• Cats that were considered non-socialised with people had higher CSS levels than those considered socialised, irrespective of housing type	• The highest reduction in CSS scores occurred between the first and fourth and first and fifth days within the boarding cattery
			• These cats also had the lowest adoption rate.			
	• Urinary cortisol: creatinine ratios were higher in singly housed cats (not statistically significant)	• No statistically significant differences between groups				
			• No significant differences noted between other housing conditions			
					• Where cats were considered socialised to other cats, CSS did not differ between single and group housing	
						• “Control” group cats had significantly lower stress levels than boarding cats
				• Overall CSS scores were higher in cats housed communally than cats housed in discrete units alone or with previously familiar conspecifics Highest scores were only seen in communal housing**		
					• Those considered non-socialised to conspecifics had higher CSS than those which were socialised to conspecifics, when housed in groups	Housing type did not appear to influence CSS in boarding cats
					• Cats considered non-socialised to conspecifics had lower CSS during the first hour of the study and on the last two days when housed singly compared with group housing	
				• Play and resting/sleeping in close contact with conspecifics were observed in more instances in cats housed in pairs or threes than in communal housing**		
					• Group housed cats had higher CSS when a cat considered non-socialised to conspecifics entered the group, compared to when a cat considered socialised with conspecifics entered	
				• Agonistic encounters were observed in more instances in communal housing than in discrete--unit housing**		
**Conclusion**	• The experience of cats being exposed to a rotation of individual cages of varying sizes for 18 consecutive days (6 days in 3 different cages) appears to be more physiologically stressing than when they are housed in a familiar group environment, with no intervention	• Group versus single housing did not result in a significant difference in cortisol: creatinine ratios	• Cats in barren single housing had higher stress levels than cats in the other 3 housing types and lowest adoption rates	• Whether cats are housed individually or with one to two other familiar conspecifics does not appear to differentially affect stress levels	• Cats which have not been previously socialised to humans may find the shelter environment more stressful than those accustomed to humans	• Cats appeared to find an established colony environment to be less stressful than any of the boarding environments, whether single or group housed with novel or familiar conspecifics
					• Cats which are not successfully socialised to conspecifics may find group housing more stressful than those socialised to conspecifics	
				• Housing cats in large groups appears to be more stressful than housing cats in discrete units (1–3 individuals in a single unit)**		
						• Suggests that the novelty of the environment may be associated with a stress response
**Main limitations**	• No sample size calculation, but sample size very small.	• No sample size calculation, but sample size very small.	• No sample size calculations	• No blinding of observer	• No sample size calculations	• No sample size calculation
			• No blinding of observer			
				• Non-random assignment of cats to groups		
						• No blinding of observer
					• No blinding of observer	
						• Comparisons made between two very different types of cats
	• No blinding of observer					
					• Randomisation method not described	
			• Insufficient detail to determine if groups were comparable at baseline			
	• Randomisation methods unclear	• No blinding of observer				
					• Sample size relatively small considering 8 sub-groups analysed	• The single and pair housing enclosures were less enriched than the group housing enclosures, which could have confounded the results
				• “Discretely-housed” cats could be housed singly, or in twos or threes with other cats they were previously socialised to. This limits the extent to which such comparisons meet the criteria of this CAT		
			• Randomisation method not specified			
	• Inappropriate comparisons used – stable enriched social group versus relatively barren single housing with minimal human contact	• No detail of how cats were assigned to groups				
			• Groups not treated equally – all but basic single received extra human interaction causing potential confound			
					• The validity of Cat-Approach and Human-Approach-Tests is questionable based on the methods used, (Non-conformity between the two different measures used to assess whether cats were socialised with conspecifics and with humans led to 30% of individuals being excluded from the data analysis)	
		• Single cortisol: creatinine measure of uncertain significance				
	• Stress measures may have related to barren environment/ frequent changes to housing conditions, especially in cats accustomed to a stable group housing situation		• Validity of the CSS as a measure of ‘Stress’ in cats (see Table 2)			
						• Excluding individuals that were ‘highly stressed’ a potential confounder
		• Singly housed cats may have had an increase in cortisol due to having had a change in environment a week previously				
			• No physiological measures considered			
						• Validity of the CSS as a measure of ‘Stress’ in cats (see Table 2)
						• No physiological measures considered
				• Total residence time of each individual within the shelter prior to study not accounted for but could have acted as a confounder if not appropriately controlled for		
					• No physiological measures considered	
				• Cats were in two different shelters - the external environment varied between each group		
				• Cat density per unit varied considerably in the discrete unit housing depending on whether there were 1,2 or 3 individuals housed together, whereas density was more consistent between communally housed groups		
				• Cats had already had one month to acclimatise to shelter environment prior to sampling – external validity		
				• Behavioural time budgets potentially a crude form of measurement to assess stress		
				• No physiological measures considered		
		• Main aims of this study were not related to the topic of the CAT				
	• Behavioural time budgets potentially a crude form of measurement to assess stress					

*The primary aims of this study were unrelated to the CAT question. However, a small sub-component of the study was relevant, and the critical review refers only to this portion.

**This portion of the study relates to comparisons that did not meet the inclusion criteria of the CAT due to the way that data was concatenated prior to statistical comparison. Such results are however included because they are considered otherwise highly relevant to the topic of the CAT

### Summary of the evidence

The findings of the appraisals are summarised in Table 1. There was a lack of agreement overall as to whether single or multi-cat housing was associated with higher levels of stress. The majority of the studies (four out of six) showed no difference in stress levels between single and multi-cat housing [30-33]. However, one of these studies only compared single cats with those housed with one or two other familiar conspecifics and not with larger multi cat groups [33]. One study suggested that stress levels were higher in cats housed singly in barren environments as compared to singly and group-housed cats provided with varying levels of enrichment [34].The final study included showed no difference in stress levels between single and group housing in socialised cats, but found that cats previously unsocialised to conspecifics showed fewer signs of stress when single housed [35].

There were significant limitations to all of the identified studies. These included differential treatment of the groups within the study. For example cats in the single housing conditions either had inconsistent handing [34], were exposed to their housing condition for a much shorter period of time [30,34], were deliberately given barren, non-enriched housing [34], or experienced a non-stable environment over the course of the study period [30], when compared with group-housed cats. Sample size calculation was performed in only one study [33], and some of the studies involved very small numbers of cats, which in one case amounted to six cats each exposed to three different interventions [30]. In none of the studies was the assessor of the outcome blinded to the intervention.

Additionally, the diverse populations under study and variations in methodology complicate comparison. Group sizes in the multi-cat environment were variable, from 2 to eight [33,34]. The effect of population density was not assessed, as this information was not available for all studies; however this may clearly be a potential confounding factor. The previous social experience of the cats varied, with some cats living in established social groups [30], some having been assessed as non-socialised to other cats by shelter staff [35] and others with no known or stated history of socialisation. A cats prior social experience was identified by one study as a factor in its stress levels in group housing, and the same study showed that the introduction of an “unsocialised” cat to a stable group caused an increase in the stress levels of all of the cats under observation [35].

There were also substantial differences in duration of the data collection periods across all studies, ranging from a single instance [31] to fifteen days [34], which could have affected the extent to which the cats had the opportunity to habituate to their respective study environments, or resulted in some cats exhibiting acute and others chronic signs of stress.

Thus these studies may not be truly comparing single and multi-cat environments, so much as suggesting the presence of several other factors that may be equally important in determining stress levels. These include: how consistent handling and husbandry routines are [34], as well as the amount of environmental manipulation, such as changes in housing location and type, that the individual is exposed to [30]. In one study, stress levels in their stable, long-term and group housed control population were lower than in any other experimental condition (i.e. individual, pair and group) [32], suggesting that group stability (and presumably familiarity) were also important mitigators of stress levels.

## Discussion

The majority of the studies did not find significant differences between single and group housed cats in regards to their stress levels. Whilst this may suggest that group size does not in fact impact upon the stress of confined cats in rehoming and similar environments, it is arguable whether this can be assumed unequivocally. This is due to the lack of overall agreement between studies, as indicated by the conflicting evidence found in two of four such studies [34,35], as well as the various confounding elements of study designs found throughout the reviewed papers. These included factors such as differential provision of enrichment or human contact between groups, differences in the cats’ socialisation and housing experience prior to the studies, and potential differences in sizes of groups in the group housing conditions. These results also suggest that a stable environment (both social and physical) may be an important factor in managing stress, and that some cats (such as those previously successfully socialised to conspecifics) may cope better in a multi-cat environment than those with little, or aversive previous experience of conspecifics. Therefore, when providing housing for cats, it is important to consider their likely prior social experience. When housing cats communally, keeping cats in large group sizes may also be more stressful than keeping them in smaller groups [33] although there is only a small amount of relevant data to support this, and it is possible that population density may also be a confounding factor.

Measuring stress in non-human animals is inherently difficult, and it is unlikely that any one measure can accurately capture how stressed an animal is [36,37]. However, the more separate (suitable) measures considered within a single study, the greater the potential for robustness. As there is no consistent definition used within the scientific literature for this term nor specific aetiology or prognosis for stress [23], it is important that where studies attempt to measure stress, a clear definition of this concept is given. This will facilitate in the ease of assessing the suitability of study methodology, as well as determining whether the main aims and objectives of a study have been achieved. All of the studies aimed to measure stress, but only one of them attempted to provide a clear definition of it [33]. Of the six papers that were critically appraised, only one study used both behavioural and physiological measures to assess stress [30] and only one used more than one set of behavioural outcome measures [33]. Only one study assessed whether the study cats were previously socialised with conspecifics [35], which again makes direct comparison between group housing conditions across the different studies difficult, because this appeared to influence the stress levels experienced by cats when housed in groups.

Comparison between the studies is further complicated by the variety of methods used to assess stress, all of which have their limitations (further details of these methods used are provided in Table 2). The duration of time over which individuals were exposed to specific housing conditions also varied considerably (both within and between studies). This affects the comparison of stress levels between cats under different housing conditions due to potential confounds of comparing cats which are acutely stressed (e.g. from being taken from stable enriched group housing to barren single housing) to cats which are chronically stressed, or to those that have actually begun to habituate to their environment. The physiological and behavioural signs of acute as compared to chronic stress may vary [38,39] making it difficult to isolate the specific effects of the environment, from the effects of period of exposure, upon the stress levels experienced by cats. However, by implication, the acutely raised stress levels in some of the single housed cats may have been as attributable to the acute change in environment rather than to the actual housing condition itself.

**Table 2:** Further detail of behavioural outcome measures used in the studies reviewed

**Measure**	**Description**	**Evidence of validity of measure**
**Cat Stress Score (CSS):**	A 7 rank linear scoring system based on key aspects of body posture and behaviour, rating cats from fully relaxed (1) to terrorised (7). Developed by Kessler and Turner [32], the CSS is a modification of the Cat Assessment Score (CAS) [40]	Evidence of correlative relationships with cortisol: creatinine ratios, but not consistent between studies [41,42], although this could be due to variation in study methodologies
		Good inter-observer reliability reported, however observer training required and there is no published training guide
		Important behaviours such as grooming are not included in the scoring system, neither are social behaviours towards conspecifics or the human observer (if they are present during scoring)
**Cat Approach Test (CAT):**	A 6 rank linear scoring system (from extremely friendly (1) to extremely unfriendly (6)) based on the response of cats to visual contact with a 4 year old male cat described as socialised with conspecifics. Cats were defined as socialized towards conspecifics when the mean of eight test ratings resulted in a score below 3.0, and non-socialized when they scored higher than 4.0. Developed by Kessler and Turner [35]	No evidence of previous attempts to validate
		No mention of inter-observer reliability
**Human Approach Test (HAT):**	A 6 rank linear scoring system (from extremely friendly (1) to extremely unfriendly (6)) based on the response of cats to a staggered human approach to their cage. Cats were defined as socialized towards people when the mean of eight test ratings resulted in a score below 3.0, and non-socialized when they scored higher than 4.0. Developed by Kessler and Turner [35], a modification of the Stranger-Approach-Test [40]	As above
**Socialisation questionnaire**	A linear scoring system based on information from multiple-choice questions (answered by the person relinquishing the cat) referring to the behavioural reactions of the cat in 10 specific situations when interacting with an unfamiliar and a familiar person, and five situations when interacting with an unfamiliar and a familiar cat. Developed by Kessler and Turner [35]	As above
**Cortisol: creatinine ratio**	Comparison of quantity of urinary cortisol with concentration of urine (as determined by quantity of creatinine present). Cortisol is an indication of physiological arousal, often used as an indirect measure of stress, although levels can vary with diurnal rhythm and other metabolic processes [41,43]	Assays based on in-house adaptation of previously validated measures [44,45]; some details not supplied in manuscript
		Evidence of correlative relationships between cortisol:creatinine ratios and CSS, but not consistent between studies [41,42], although this could be due to variation in study methodologies
		Evidence of correlative relationships between cortisol concentrations and the exposure to environmental stressors [43]
		Evidence that cortisol levels do not necessarily correlate with other physiological indicators of stress or compromised immunity [46]

Whether individuals have previous experience of the housing environment may also be another important mitigator of stress. Previous research indicates that cats that have been housed in rehoming centre environments previously may cope better under these conditions than those that have not [47]. It is unknown if any of the study populations within the appraised papers had been housed under such conditions before, but this may have been an important factor to consider.

## Conclusion

On the basis of the evidence available, the below recommendations for practice have been provided. It is however important to consider the complex nature of stress, and the methodological limitations of the above studies, in relation to their ability to help us isolate and assess the effects of multiple and single housing alone on stress in cats (Table 1). There are also numerous other factors which have not been considered here, particularly disease control, which is also of great importance in rehoming centres [48-50]. These results should draw attention to the importance of other potential mitigating factors which may influence how stressful single or multiple housing can be for individuals, and suggest ways these may be utilised practically to improve the welfare of confined cats in these types of environments.

### Recommendations for practice

• Especially where the previous social history of cats towards conspecifics is unknown, individuals should be housed singly, but with the appropriate environmental enrichment in place (e.g. places to hide and perch, toys, consistent positive human handling where appropriate).

• Cats should be exposed to as few environmental changes/manipulations as possible during their stay and husbandry routines should be as consistent as possible.

• If cats are to be housed in groups, they should ideally be housed together with other cats considered socialised to conspecifics.

• If cats are to be housed in groups, or with those that are initially unfamiliar, wherever possible, groups should have a stable composition (i.e. group members are not constantly changed).

## Competing interests

The authors declare that they have no competing interests

## Authors’ contributions

LF carried out data collection, coordination, analysis and interpretation of data as well as the drafting of the manuscript. SE contributed towards the analysis and interpretation of data as well as critical revision of the manuscript. JS conceived of the study, participating in its design and coordination, data collection and critical revision of the manuscript. All authors read and approved the final manuscript.

## Supplementary Material

Additional file 1 Search terms used in constructing the CAT. Click here for file

## References

[B1] McCune S Smith CP Taylor V Nicol C Enriching the environment of the laboratory cat Environmental Enrichment Information Resources for Laboratory Animals: 1965–1995 Birds, Cats, Dogs, Farm Animals, Ferrets, Rabbits, and Rodents 1996 DIANE Publishing 27 42

[B2] Rochlitz I Recommendations for the housing of cats in the home, in catteries and animal shelters, in laboratories and in veterinary surgeries J Feline Med Surg 1999 1 3 181 191 10.1016/S1098-612X(99)90207-3 11919033 PMC10832797

[B3] Rochlitz I Recommendations for the housing and care of domestic cats in laboratories Lab Anim 2000 34 1 1 9 10.1258/002367700780577939 10759361

[B4] Stavisky J Brennan M Downes M Dean R Demographics and economic burden of un-owned cats and dogs in the UK: results of a 2010 census BMC Vet Res 2012 8 1 1 10 10.1186/1746-6148-8-1 22974242 PMC3514250

[B5] Serpell JA Domestication and History of the Cat The Domestic Cat: The Biology of its Behaviour 2000 2 Cambridge: Cambridge University Press 179

[B6] Randi E Ragni B Genetic Variability and Biochemical Systematics of Domestic and Wild Cat Populations (Felis silvestris: Felidae) J Mammal 1991 72 1 79 88 10.2307/1381981

[B7] Johnson W O’Brien S Phylogenetic reconstruction of the felidae using 16S rRNA and NADH-5 mitochondrial genes J Mol Evol 1997 44 1 S98 S116 10.1007/PL00006127 9071018

[B8] Page R Ross J Bennet D A study of the home ranges, movements and behaviour of the feral cat population at Avonmouth Docks Wildl Res 1992 19 3 263 277 10.1071/WR9920263

[B9] Genovesi P Besa M Toso S Ecology of a feral cat Felis catus population in an agricultural area of northern Italy Wildl Biol 1995 1 4 233 237

[B10] Fitzgerald BM Karl BJ Home range of feral house cats (Felis catus) in forest of the Orongorongo Valley, Wellington, New Zealand N Z J Ecol 1986 9 71 82

[B11] Devillard S Say L Pontier D Dispersal pattern of domestic cats (Felis catus) in a promiscuous urban population: do females disperse or die? J Anim Ecol 2003 72 2 203 211 10.1046/j.1365-2656.2003.00692.x

[B12] Macdonald DW Yamaguchi N Kerby G Group-living in the Domestic Cat: Its Sociobiology and Epidemiology The Domestic Cat: The Biology of its Behaviour 2000 2 2 Cambridge: Cambridge University Press 95 118

[B13] Turner DC Mertens C Home range size, overlap and exploitation in domestic farm cats (Felis Catus) Behaviour 1986 99 1–2 22 45

[B14] Natoli E Spacing pattern in a colony of urban stray cats (Felis catus L.) in the historic centre of Rome Appl Anim Behav Sci 1985 14 3 289 304 10.1016/0168-1591(85)90009-7

[B15] Mirmovitch V Spatial organisation of urban feral cats (Felis Catus) in Jerusalem Wildl Res 1995 22 3 299 310 10.1071/WR9950299

[B16] Liberg O Sandell M Pontier D Natoli E Density, spatial organisation and reproductive tactics in the domestic cat and other felids The Domestic Cat: The Biology of its Behaviour 2000 2 Cambridge: Cambridge University Press 119 147

[B17] Say L Pontier D Spacing pattern in a social group of stray cats: effects on male reproductive success Anim Behav 2004 68 1 175 180 10.1016/j.anbehav.2003.11.008

[B18] Bradshaw JWS Hall SL Affiliative behaviour of related and unrelated pairs of cats in catteries: a preliminary report Appl Anim Behav Sci 1999 63 3 251 255 10.1016/S0168-1591(99)00007-6

[B19] van den Bos R Post-conflict stress-response in confined group-living cats (Felis silvestris catus) Appl Anim Behav Sci 1998 59 4 323 330 10.1016/S0168-1591(98)00147-6

[B20] Tanaka A Wagner DC Kass PH Hurley KF Associations among weight loss, stress, and upper respiratory tract infection in shelter cats J Am Vet Med Assoc 2012 240 5 570 576 10.2460/javma.240.5.570 22332626

[B21] Ward PA Blanchard RJ Bolivar V Recognition and Alleviation of Distress in Laboratory Animals 2008 Washington (DC): The National Academies Press 20669418

[B22] McEwen BS Wilson RA, Keil F Stress The MIT Encyclopedia of the Cognitive Sciences 1999 Cambridge, Mass: MIT Press

[B23] Moberg GP Biological response to stress: implications for animal welfare The Biology of Animal Stress: Basic Principles and Implications for Animal Welfare 2000 1 21

[B24] Dohms JE Metz A Stress — mechanisms of immunosuppression Vet Immunol Immunopathol 1991 30 1 89 109 10.1016/0165-2427(91)90011-Z 1781159

[B25] Blood DC Radostits OM Veterinary medicine : a textbook of the diseases of cattle, sheep, pigs, goats and horses 2000 London: W. B. Saunders

[B26] Liberati A Altman DG Tetzlaff J Mulrow C Gøtzsche PC Ioannidis JPA Clarke M Devereaux PJ Kleijnen J Moher D The PRISMA statement for reporting systematic reviews and meta-analyses of studies that evaluate healthcare interventions: explanation and elaboration BMJ 2009 339 b2700 10.1136/bmj.b2700 19622552 PMC2714672

[B27] Garritsen FM ter Haar NM Spuls PI House dust mite reduction in the management of atopic dermatitis. A critically appraised topic Br J Dermatol 2013 168 4 688 691 10.1111/bjd.12283 23528056

[B28] Guinane S The effectiveness of probiotics for managing diarrhoea in people with HIV infection: a critically appraised topic HIV Med 2013 14 187 190 10.1111/j.1468-1293.2012.01042.x 22989042

[B29] Nishijima DK Zehtabchi S Evidence-based Emergency Medicine/Critically appraised topic. The efficacy of recombinant activated factor VII in severe trauma Ann Emerg Med 2009 54 5 737 10.1016/j.annemergmed.2009.01.027 19285753

[B30] Uetake K Goto A Koyama R Kikuchi R Tanaka T Effects of single caging and cage size on behavior and stress level of domestic neutered cats housed in an animal shelter Anim Sci J 2013 84 3 272 274 10.1111/j.1740-0929.2012.01055.x 23480709

[B31] Lichtsteiner M Turner DC Influence of indoor-cat group size and dominance rank on urinary cortisol levels Anim Welf 2008 17 3 215 237

[B32] Kessler MR Turner DC Stress and adaptation of cats (Felis silvestris catus) housed singly, in pairs and in groups in boarding catteries Anim Welf 1997 6 3 243 254

[B33] Ottway DS Hawkins DM Cat housing in rescue shelters: a welfare comparison between communal and discrete-unit housing Anim Welf 2003 12 2 173 189

[B34] Gourkow N Fraser D The effect of housing and handling practices on the welfare, behaviour and selection of domestic cats (Felis sylvestris catus) by adopters in an animal shelter Anim Welf 2006 15 4 371

[B35] Kessler MR Turner DC Socialization and stress in cats (Felis silvestris catus) housed singly and in groups in animal shelters Anim Welf 1999 8 1 15 26

[B36] Dawkins MS Animal Suffering: The Science of Animal Welfare 1980 London: Chapman and Hall Ltd

[B37] Mason G Mendl M Why is there no simple way of measuring animal welfare? Anim Welf 1993 2 4 301 319

[B38] Rostamkhani F Zardooz H Zahediasl S Farrokhi B Comparison of the effects of acute and chronic psychological stress on metabolic features in rats J Zhejiang Univ Sci B 2012 13 11 904 912 10.1631/jzus.B1100383 23125083 PMC3494029

[B39] Dhabhar FS McEwen BS Acute stress enhances while chronic stress suppresses cell-mediated immunity in vivo: a potential role for leukocyte trafficking Brain Behav Immun 1997 11 4 286 306 10.1006/brbi.1997.0508 9512816

[B40] McCune S Temperament and the Welfare of Caged Cats Doctoral dissertation 1992 Cambridge: University of Cambridge

[B41] McCobb EC Patronek GJ Marder A Dinnage JD Stone MS Assessment of stress levels among cats in four animal shelters J Am Vet Med Assoc 2005 226 4 548 555 10.2460/javma.2005.226.548 15742695

[B42] Hawkins KR Stress, Enrichment and the Welfare of Domestic Cats in Rescue Shelters Doctoral Dissertation 2005 Bristol: University of Bristol

[B43] Carlstead K Brown JL Strawn W Behavioral and physiological correlates of stress in laboratory cats Appl Anim Behav Sci 1993 38 2 143 158 10.1016/0168-1591(93)90062-T

[B44] Bahr NI Pryce C Dobeli M Martin RD Evidence from urinary cortisol that maternal behavior is related to stress in gorillas Physiol Behav 1998 64 4 429 437 10.1016/S0031-9384(98)00057-2 9761215

[B45] Dettling A Pryce CR Martin RD Döbeli M Physiological responses to parental separation and a strange situation are related to parental care received in juvenile Goeldi’s monkeys (Callimico goeldii) Dev Psychobiol 1998 33 1 21 31 10.1002/(SICI)1098-2302(199807)33:1<21::AID-DEV3>3.0.CO;2-U 9664169

[B46] Gourkow N LaVoy A Dean GA Phillips CJC Associations of behaviour with secretory immunoglobulin A and cortisol in domestic cats during their first week in an animal shelter Appl Anim Behav Sci 2014 150 55 64

[B47] McCune S Caged cats: avoiding problems and providing solutions Newsletter of the Companion Animal Study Group 1994 7 1 9

[B48] Bannasch MJ Foley JE Epidemiologic evaluation of multiple respiratory pathogens in cats in animal shelters J Feline Med Surg 2005 7 2 109 119 10.1016/j.jfms.2004.07.004 15771947 PMC10822251

[B49] Carlotti DN Guinot P Meissonnier E Germain PA Eradication of feline dermatophytosis in a shelter: a field study Vet Dermatol 2010 21 3 259 266 10.1111/j.1365-3164.2009.00789.x 19706005

[B50] Coyne KP Edwards D Radford AD Cripps P Jones D Wood JLN Gaskell RM Dawson S Longitudinal molecular epidemiological analysis of feline calicivirus infection in an animal shelter: a model for investigating calicivirus transmission within high-density, high-turnover populations J Clin Microbiol 2007 45 10 3239 3244 10.1128/JCM.01226-07 17687017 PMC2045375

