# Supplementary figures and images for: A critically appraised topic (CAT) to compare the effects of single and multi-cat housing on physiological and behavioural measures of stress in domestic cats in confined environments
Source: BMC Vet Res. 2014 Mar 22;10:73. doi: 10.1186/1746-6148-10-73 (PMC3998042; doi:10.1186/1746-6148-10-73)

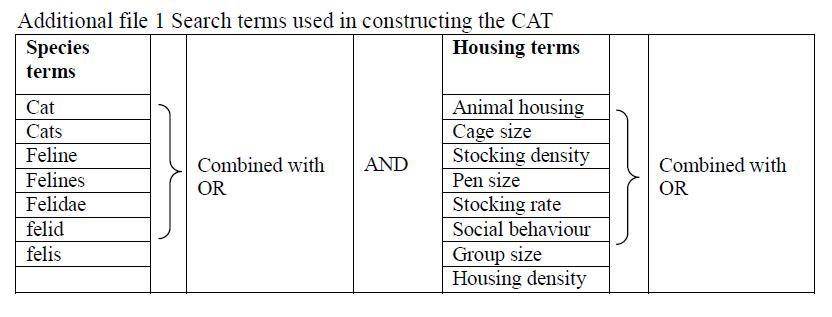

Supplement: Additional file 1 — Search terms used in constructing the CAT. [file 1746-6148-10-73-S1.jpg]
